# Prognostic Significance of Pretreatment Neutrophil/Lymphocyte Ratio and Platelet/Lymphocyte Ratio in Patients with Diffuse Large B-Cell Lymphoma

**DOI:** 10.1155/2018/9651254

**Published:** 2018-12-12

**Authors:** Siqian Wang, Yongyong Ma, Lan Sun, Yifen Shi, Songfu Jiang, Kang Yu, Shujuan Zhou

**Affiliations:** ^1^Department of Prosthodontics, School & Hospital of Stomatology Wenzhou Medical University, Wenzhou, Zhejiang 325000, China; ^2^Department of Hematology, The First Affiliated Hospital of Wenzhou Medical University, Wenzhou, Zhejiang 325000, China

## Abstract

It is generally believed that there is correlation between cancer prognosis and pretreatment PLR and NLR. However, there are limited data about their role in diffuse large B cell lymphoma (DLBCL). This study aims to determine the prognostic value of pretreatment PLR and NLR for patients who have DLBCL. The associations between clinical characteristics and NLR and PLR were evaluated among 182 DLBCL patients from January 2005 to June 2016. The optimal cutoff values for high PLR (*⩾*150) and NLR (*⩾*2.32) in prognosis prediction were determined. The effect of NLR and PLR on survival was evaluated through multivariate Cox regression analysis, univariate analysis, and log-rank test. According to the evaluation results, patients with high NLR and PLR had significantly shorter OS (P=0.026 and P=0.035) and PFS (P=0.024 and P=0.022) compared with those who have low PLR and NLR. On multivariate analyses, IPI>2, elevated LDH, and PLR*⩾*2.32 were prognostic factors for OS and PFS in DLBCL patients. Therefore, we demonstrated that high PLR and NLR predicted adverse prognostic factors in DLBCL patients.

## 1. Introduction

With a prevalence of about 25% non-Hodgkin's lymphomas (NHL) [1], diffuse large B-cell lymphoma (DLBCL) is a common lymphomas group. This malignancy is a heterogeneous entity, including variable clinical behaviors, morphologic variants, various biologic abnormalities, and different responses to treatment [2]. Some prognostic factors, such as gene expression profiling (GEP) [3], early interim analysis with positron emission tomography after chemotherapy [4, 5], and International Prognostic Index (IPI) [6], have been studied. However, the treatment for some patients who have favorable prognostic factors fails and vice versa.

In recent years, increasing attention has been attached to the correlation between cancer and inflammation. There is antitumor activity in inflammation and activation of immune system, which promotes tumor growth, human cancer progression, and carcinogenesis [7]. Systemic inflammation is an independent risk factor for treatment response, overall survival (OS), and event-free survival in patients with DLBCL [8–10].

Platelet/lymphocyte ratio (PLR) and pretreatment neutrophil/lymphocyte ratio (NLR) are common clinical parameters of inflammation and blood system, and they are related to the adverse prognosis in several malignancies [11–14]. However, the use of PLR and NLR as a prognostic marker for DLBCL is seldom reported [15–17]. Therefore, this study aims to explore the potential prognostic function of NLR and PLR in DLBCL by comparing the pretreatment complete blood count (CBC) data of patients with other risks of mortality.

## 2. Materials and Methods

### 2.1. Patients and Methods

The inclusion criteria are diagnosed de novo DLBCL, treated with R-CHOP (prednisone, hydroxydaunorubicin, vincristine, cyclophosphamide, and rituximab) or R-chop-like chemotherapy for a minimum of 4 cycles in which the clinical data are complete, and follow-up at the First Affiliated Hospital of Wenzhou Medical University between January 2005 and June 2016. The patients with HIV associated DLBCL, transformed NHL, or inflammatory conditions, including collagen diseases or infections, or other diseases of the hematological system, pretreatment with induction radiotherapy or chemotherapy, as well as a previous malignancy or non-cancer-associated death were excluded. From January 2005 to June 2016, 182 DLBCL patients were enrolled in the study, which was performed according to the principles of the Declaration of Helsinki, which had obtained the approval of the Institutional Review Board of our hospital.

### 2.2. Clinical Data

The clinicopathologic characteristics and demographic data of patients and were collected from medical records, which include International Prognostic Index (IPI), LDH, pathology type, ECOG-PS, and the presence of B symptoms.

Lymphocyte, neutrophil, and platelet counts were obtained from standard CBC data before the initiation of any treatment (pretreatment). NLR was calculated using the formula absolute neutrophil count which was divided through absolute lymphocyte count. PLR was calculated as the absolute platelet count which was divided through absolute lymphocyte count. ROC curves were used to determine the best threshold values for sensitivity and specificity.

Cheson BD et al. defined the relapse and response criteria [18]. PFS was defined as the time from diagnosis to relapse. And OS was from diagnosis to death.

### 2.3. Statistical Analysis

IBM SPSS Statistic v. 21.0 (SPSS Inc., Chicago, IL, USA) was used to perform statistical analysis. The categorical variables were demonstrated according to the frequency (%). The categorical variables were analyzed through Chi-square/Fisher's exact test. Prognostic factors of OS and PFS were determined through univariate and multivariate Cox regression analyses. Variables with P<0.05 in univariate analysis were introduced to a multivariate analysis. Two-tailed P-values were reported. P<0.05 was considered of statistical significance.

## 3. Results

The enrollment was carried out among 182 patients. The median follow-up time was 24 months (6-120 months). 59 (range 18-80) years was the median age.  Table 1 shows other clinical and laboratory characteristics of the patients who had been dead by the time of the last follow-up. The median time of death was 22 (7-89) months. Their death resulted from lymphoma relapse by the time of the last follow-up.

**Figure 1:**
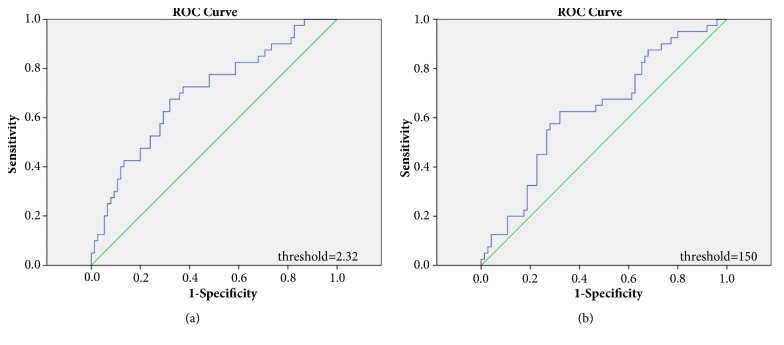
ROC curves analysis for NLR (a) and PLR (b).

The optimal cutoff values of pretreatment PLR and NLR were determined through ROC analysis. The area under the ROC curves for NLR [Figure 1(a)] and PLR [Figure 1(b)] was 0.698 (95% CI: 0.596–0.799, P <0.001) and 0.631 (95% CI: 0.524–0.737, P=0.021), respectively (Figure 1). According to the ROC curve, the optimal cutoff levels were 2.32 for NLR and 150 for PLR.

There were 96 patients with NLR*⩾*2.32 and 86 patients with NLR<2.32.  Table 1 shows the correlation between NLR and the clinical factors. The patients who have high NLR showed a higher disease stage (P=0.001) more frequently, with B symptoms (P=0.005), and had significantly lower ECOG-PS (P=0.048), higher LDH (P=0.006), more extranodal sites of disease (P=0.002), and higher IPI (P=0.001) at diagnosis. NLR is not significantly related to clinical factors, such as gender, age, bone marrow infiltration, and pathology type (Table 1).

There were 92 patients with PLR<150 and 90 patients with PLR*⩾*150. The associations between PLR and the clinical factors are shown in Table 1. The patients with high PLR have lower ECOG-PS (P=0.032), with B symptoms (P=0.039), and had a higher LDH (P=0.006) at diagnosis. PLR was not related to other clinical features, including disease stage, age, IPI, gender, pathology type, extranodal sites of disease, and bone marrow infiltration (Table 1).

**Figure 2:**
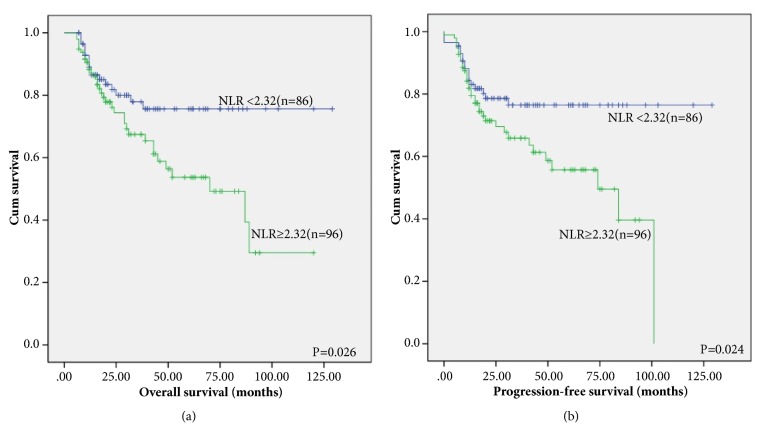
Kaplan–Meier survival analysis of NLR. OS (a) and PFS(b) according to NLR in DLBCL patients.

Through Kaplan-Meier analysis, whether NLR and PLR were associated with PFS and OS was valued. The group with a NLR<2.32 has shorter OS and PFS compared to those in the group with a NLR≥2.32 [Figure 2(a), P=0.026; Figure 2(b), P=0.024]. In the NLR<2.32 group, the 2-year OS rates were 83.7, but in the NLR≥2.32 group, they were 78.1 [Figure 2(a)]. Correspondingly, the 2-year PFS rates separately were 80.0 in the NLR<2.32 group. However, the PFS rate of the NLR≥2.32 group was 74.0 [Figure 2(b)].

**Figure 3:**
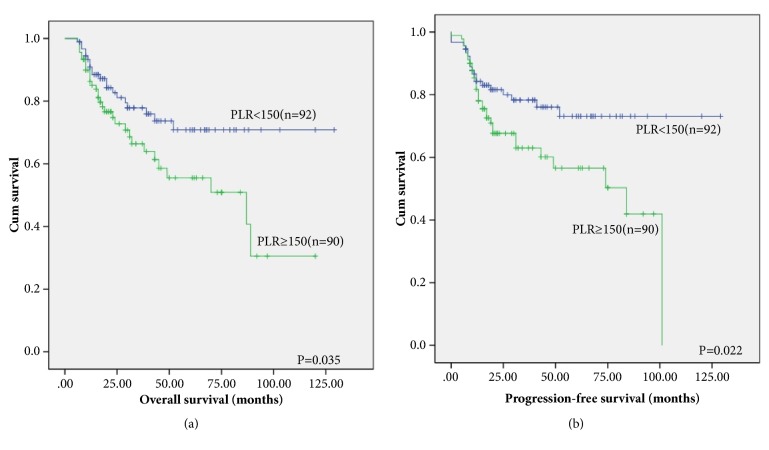
Kaplan–Meier survival analysis of PLR. OS (a) and PFS (b) according to PLR in DLBCL patients.

According to Kaplan-Meier curves, the patients who have low PLR experienced superior PFS [Figure 3(b), P=0.022] and OS [Figure 3(a), P=0.035] than the patients with a high PLR. The two-year OS rates were 84.8% in the PLR<150 group and 76.7% in the PLR≥150 group [Figure 3(a)]. Correspondingly, the two-year PFS rates were 82.6% in the PLR<150 group and 71.1% in the PLR≥150 group [Figure 3(b)].

Tables 2 and 3 summarized the univariate and multivariate analysis results of clinical factors that influence PFS and OS in DLBCL patients.

By carrying out univariate Cox regression analysis, it can be found that independent predictors of OS were a higher clinical stage (P=0.025), a higher ECOG-PS (P=0.007), higher IPI (P=0.001), with B symptoms (P=0.023), elevated LDH (P=0.001), more extranodal sites of disease (P=0.010), higher NLR (P=0.030), and elevated PLR (P=0.038). Independent predictors of PFS had higher clinical stage (P=0.047), higher ECOG-PS (P=0.024), more extranodal sites of disease (P=0.025), with B symptoms (P=0.021), higher IPI score (P=0.002), elevated LDH (P=0.001), high NLR (P=0.028), and elevated PLR (P=0.025) (Table 2).

Multivariate analyses were performed through the Cox proportional hazard model, including all parameters that are significant at P<0.05. Neutrophils is a type of inflammatory cells, and thrombocytes are present as a nonspecific response to inflammation induced by cancer. Due to the correlation between NLR and PLR, two multivariate analyses will be carried out separately, one with NLR and one with PLR to avoid the problem with colinearity. The results revealed that PLR*⩾*2.32 (P=0.038; P=0.041), LDH>ULN (P=0.046; P=0.039), and IPI>2 (P=0.041; P=0.044) were independent prognostic factors for PFS and OS in DLBCL patients in PLR model (Table 3). In NLR model, the results revealed only that LDH>ULN (P=0.046; P=0.044) and IPI>2 (P=0.039; P=0.046) were independent prognostic factors for PFS and OS in DLBCL patients (Table 4).

## 4. Discussion

It is commonly recognized that inflammation plays a very significant role in the development of cancer and may affect cancer patients' survival [19]. Systemic inflammation promotes tumor metastasis and progression through the promotion of angiogenesis, apoptosis inhibition, and DNA damage [20]. It has been found that hematological indices for these systemic inflammatory conditions, such as platelet count, NLR and PLR, and leukocyte count, are independent prognostic factors in patients with various cancer types [21–23]. But whether NLR and PLR are associated with prognosis in hematological malignancy is under strong research interest. Only little research investigated pretreatment NLR's prognostic role in people suffering from DLBCL [17, 24, 25], and the utility of PLR in DLBCL patients remains unknown.

In this study, we found that the patients with higher NLR and PLR had markedly short OS and PFS compared with those with low NLR and PLR. The patients with a high NLR more frequently showed significantly lower ECOG-PS, had a higher disease stage, with B symptoms, and more extranodal sites of disease, and had a higher IPI and a higher LDH at diagnosis. The patients with a high PLR more frequently showed significantly lower ECOG-PS, with B symptoms, and had a higher LDH at diagnosis. Multivariate analyses also showed high PLR to be an independent prognostic factor for mortality in DLBCL.

Although cancer and inflammation have been strongly linked with each other, the mechanism between increased NLR and PLR and poor tumor prognosis is still unclear. Tumor-associated inflammatory responses consist of a series of inflammatory mediators and inflammatory cells. Together, these generate a tumor-related inflammatory microenvironment, which plays vital roles in tumor progression and pathogenesis [26]. Furthermore, such factors may lead to decreased sensitivity of antitumor therapy. On the other hand, tumor-related inflammatory responses can result in changes in blood components such as platelets, lymphocytes, and neutrophils [27, 28].

Lymphocytes are of importance for NHL patients' immune surveillance. A number of studies demonstrated a relationship between lymphocytopenia and poor clinical outcome in NHL of various subtypes, including DLBCL [29–31]. Inhibitory immunologic cytokines such as transforming growth factor-b and IL-10, which is associated with systemic inflammation, can lead to significant immunosuppressive effects with consequent impaired cytolytic activity of the lymphocytes [32].

Neutrophils as a type of inflammatory cells promote tumor progression by producing a series of inflammatory factors that can inhibit apoptosis, promote angiogenesis, and damage cellular DNA [33–35]. Generating neutrophil extracellular traps caused by the elevated level of peripheral blood neutrophils had been reported to contribute to cancer-related thrombosis in breast and lung cancer models [36].

The rationale of NLR is to compare inflammatory responses of host (neutrophils) with the cancer that has the immune responses of host (lymphocytes). In recent years, it has been shown that NLR at diagnosis is one prognostic factor in DLBCL patients receiving R-chemotherapy [37]. In our study, we found that the patients with an NLR<2.32 at diagnosis experienced more superior PFS and OS than those within an NLR*⩾*2.32 at diagnosis. Nevertheless, we did not find any statistical significance in multivariate analysis, which may be because one type-II error is secondary to comparatively modest size of sample as well as the low event rate within the two categories. To clearly determine if NLR could improve present established risk stratification systems' prognostic value, further evidence within research with one bigger sample size is required.

Inflammatory cytokines released by various cancer entities, like IL-3, IL-6, and IL-10, are capable of stimulating megakaryocytes proliferation, which can produce platelets [38, 39]. Hence, the presence of the thrombocytosis may be one nonspecific response towards cancer-related inflammation. Recent clinical and experimental research reveals that platelet activation in the circulatory system contributes to the metastasis of tumors through growth factors that the platelets activated secret and then protects tumor cells from being attacked by immune system, promotes tumor cells for arresting in endothelium, and enhances tumor motility and growth [40–42]. Moreover, it had been reported that PLR is one prognostic marker for a number of cancers [43, 44].

Consistent with this finding, we found that patients with a PLR<150 at diagnosis experienced more superior PFS and OS than those with a PLR*⩾*150 at diagnosis. PLR predictive ability was also confirmed in our multivariate and univariate analysis, indicating that PLR as one prognostic factor for DLBCL patients may be more superior to NLR. Our finding was similar to the previous study [15, 16]. We assumed that the findings might be because the platelets are less stable and more resistant inflammatory markers than neutrophils; thus, the findings could better reflect the real response of host towards cancer-related inflammation [45].

NLR and PLR are calculated from blood cell count, which is routinely recorded in nearly all oncology records, an easily available measure in daily clinical practice, and is inexpensive to test, which can provide useful prognostic information for the management and treatment of DLBCL. Therefore, these markers can be easily accepted by clinicians.

We acknowledge that our study has certain limitations. Firstly, this study is one single-center retrospective study. Secondly, the sample size is small. Thirdly, the treatment duration of patients is relatively long. Therefore, it may lead to many biases. Hence, a multicenter and wider retrospective study should be designed for supporting the preliminary results.

In conclusion, this study demonstrated that a high NLR and PLR were associated with shorter OS and PFS. They are inexpensive instruments which are helpful to predict the outcomes of patients with DLBCL receiving R-chemotherapy. Nevertheless, further research is still needed for investigating the role of NLR and PLR among patients based on larger sample size.

## Figures and Tables

**Table 1:** Peripheral NLR/PLR and clinical characteristics of DLBCL patients.

Characteristics	Total (n=182)	PLR			NLR		
		*⩾*150(n=90)	<150(n=92)	P-value	*⩾*2.32(n=96)	<2.32(n=86)	P-value
Gender				0.659			0.883
Male, n (%)	96	49	47		50	46	
Age				0.883			0.235
>60	90	44	46		43	47	
⩽60	92	46	46		53	39	
Ann Arbor stage, n (%)				0.431			0.001
I	43	21	22		13	30	
II	61	26	35		28	33	
III	19	9	10		12	7	
IV	59	34	25		43	16	
B symptoms, n (%)				0.039			0.005
Yes	35	23	12		26	9	
No	147	67	80		70	77	
ECOG PS, n (%)				0.032			0.048
<2	142	64	78		69	73	
*⩾*2	40	26	14		27	13	
Extranodal sites of disease, n (%)				0.285			0.002
>1	40	23	17		30	10	
⩽1	142	67	75		66	76	
IPI, n (%)				0.053			0.001
0	37	16	21		16	21	
1	55	20	35		18	37	
2	38	21	17		24	14	
3	30	18	12		23	7	
4	14	11	3		10	4	
5	8	4	4		5	3	
LDH, n (%)				0.006			0.006
⩽1 × ULN	112	46	66		50	62	
>1 × ULN	70	44	26		46	24	
Bone marrow involvement, n (%)				0.567			1.000
YES	13	5	8		7	6	
NO	169	85	84		89	80	
Pathology type				0.728			0.728
GCB subtype	43	20	23		24	19	
Non-GCB subtype	139	70	69		72	67	

*∗* Serum LDH level >ULN (upper limit of normal), the normal range of LDH in our center is 0-250U/L.

Abbreviations: ECOG, Eastern Cooperative Oncology Group; PS, performance status; IPI, International Prognostic Index; LDH, lactate dehydrogenase; GCB, germinal center B cell.

**Table 2:** Univariate analysis of clinical factors for PFS and OS in 182 patients.

Characteristics	OS			PFS		
	HR	95%CI	P value	HR	95%CI	P value
Age (>60)	0.862	0.497-1.496	0.597	0.840	0.489-1.443	0.528
Gender	0.923	0.531-1.602	0.775	0.918	0.534-1.576	0.755
B symptoms	0.504	0.279-0.911	0.023	0.500	0.278-0.900	0.021
ECOG PS (*⩾*2)	0.427	0.231-0.788	0.007	0.497	0.271-0.912	0.024
LDH (>ULN)	0.405	0.232-0.707	0.001	0.385	0.222-0.667	0.001
Stage (III and IV)	0.527	0.301-0.921	0.025	0.574	0.331-0.993	0.047
Bone marrow involvement	2.227	0.945-5.252	0.067	0.506	0.215-1.191	0.119
IPI (>2)	0.379	0.216-0.665	0.001	0.420	0.241-0.732	0.002
Extranodal sites of disease (>1)	0.451	0.247-0.824	0.010	0.507	0.280-0.919	0.025
Pathology type	0.895	0.447-1.789	0.753	0.830	0.416-1.656	0.598
NLR*⩾*150	1.909	1.066-3.418	0.030	0.527	0.298-0.932	0.028
PLR*⩾*2.32	0.551	0.314-0.969	0.038	0.528	0.302-0.924	0.025

Abbreviations: NLR, neutrophil/lymphocyte ratio; PLR, platelet/lymphocyte ratio.

**Table 3:** Multivariate analysis of clinical factors for PFS and OS according to PLR.

Characteristics	OS			PFS		
	HR	95%CI	P value	HR	95%CI	P value
B symptoms	1.270	0.649-2.483	0.485	1.471	0.758-2.855	0.254
ECOG PS (*⩾*2)	0.753	0.234-2.423	0.635	0.711	0.222-2.277	0.566
LDH (>ULN)	1.704	0.719-4.036	0.046	1.895	0.813-4.419	0.039
Stage (III and IV)	0.823	0.350-1.934	0.655	0.801	0.339-1.895	0.614
IPI (>2)	1.252	0.707-2.217	0.041	1.168	0.659-2.070	0.044
Extranodal sites of disease (>1)	1.411	0.526-3.781	0.494	1.412	0.524-3.806	0.495
PLR*⩾*2.32	1.549	0.868-2.763	0.038	1.458	0.818-2.597	0.041

**Table 4:** Multivariate analysis of clinical factors for PFS and OS according to NLR.

Characteristics	OS			PFS		
	HR	95%CI	P value	HR	95%CI	P value
B symptoms	1.302	0.669-2.535	0.437	1.494	0.772-2.892	0.233
ECOG PS (*⩾*2)	0.690	0.213-2.237	0.537	0.661	0.204-2.146	0.491
LDH (>ULN)	1.701	0.720-4.015	0.046	1.879	0.807-4.377	0.044
Stage (III and IV)	0.700	0.297-1.649	0.415	0.697	0.295-1.648	0.411
IPI (>2)	1.326	0.743-2.366	0.039	1.223	0.685-2.181	0.046
Extranodal sites of disease (>1)	1.293	0.483-3.461	0.609	1.314	0.489-3.532	0.588
NLR*⩾*150	1.564	0.853-2.867	0.148	1.527	0.830-2.809	0.173

## Data Availability

The data used to support the findings of this study are available from the corresponding author upon request.
